# Utility of Carnrick’s Soluble Food

**Published:** 1887-06

**Authors:** 


### *UTILITY OF CARNRICK'S SOLUBLE FOOD.*

Dr. Malacrida, in *Gazette Degli Ospitale*, Milan, 1886.—In July last there came under my care a lady suffering from parenchymatous rheumatic nephritis. The attack was so acute as to lead to the suspicion of Variola, shivering frequent and terrible lumbar pains, vomiting, but on the third day occurred an œdema, which became general, the urine being examined, removed every doubt as to the diagnosis. In quantity half a litre in twenty-four hours; re-action acid; specific gravity 1035; reddish brown color on account of the presence of blood. Tested for albumen it became a solid brown mass, almost black. The microscope revealed a great quantity of renal epithelium, numerous blood corpuscles, both red and white, etc. The patient was threathened with pulmonary œdema, an obstinate vomiting of mucous and acid rendered impossible the administration of any remedy to overcome the constipation afflicting the patient from the first, and which prevented any possibility, by the intestinal tract, of compensating for the insufficiency of alimentation. Perspiration was induced, cups were applied to the lumbar region, attempts were made to administer milk; first pure, then deprived of butter by beating, and seasoned with salt or with Carlsbad water, but all were abandoned on account of distressing gastric symptoms being aggravated in the most extraordinary manner. Not knowing where to turn for a dietetic and curative method, it occurred to me that when sick infants were afflicted with gastro-enteritis of the gravest nature, I had always used the Carnrick Soluble Food with the greatest success. I therefore resolved to try it in this instance. I had a teaspoonful boiled a few moments in a glass of water and gave it hot to my patient, who supported it exceedingly well. I repeated the portion after a few hours. In short, nourished with this food alone and treated with a few subcutaneous injections of hydrochlorate of pilo-carpine, the gastric intolerance diminished, the tongue became clean, the breath less offensive, the quantity of albumen contained in the urine being less, mophologic elements disappeared, phosphites and chlorides re-appeared, and the patient made good recovery, not abandoning, however, for two weeks more, Soluble Food (*la sua farina alimentarè*).

The same tolerance for this nutriment was afterwards noted in two other cases of nephritis when milk could not be retained. I also attempted the administration of this food in two cases of ulcer of the stomach, but with less happy results, though the tolerance was better than for milk, especially with the addition of a strong infusion of coca or a few centigrammes of hydrochlorate of cocaine.

Another female patient afflicted with carcinoma of the pylorus, and for whom the question of alimentation was a most serious one, as she tolerated neither milk nor broth, nor extract of meat, nor peptones, supported very well the Soluble Food cooked in water, to which was added a little diluted spirit of cider.

In conclusion, I believe that of all the special foods which are more or less highly praised and introduced to our notice, this Carnrick Food merits the preference.

